# Climate alters the movement ecology of a non‐migratory bird

**DOI:** 10.1002/ece3.8869

**Published:** 2022-04-23

**Authors:** Landon K. Neumann, Samuel D. Fuhlendorf, Craig D. Davis, Shawn M. Wilder

**Affiliations:** ^1^ 7618 Oklahoma State University Stillwater Oklahoma USA; ^2^ 7618 Natural Resource Ecology and Management Oklahoma State University Stillwater Oklahoma USA; ^3^ 7618 Department of Integrative Biology Oklahoma State University Stillwater Oklahoma USA

**Keywords:** animal behavior, global climate change, movement, Northern Bobwhite, solar radiation, temperature

## Abstract

Global climate change is causing increased climate extremes threatening biodiversity and altering ecosystems. Climate is comprised of many variables including air temperature, barometric pressure, solar radiation, wind, relative humidity, and precipitation that interact with each other. As movement connects various aspects of an animal's life, understanding how climate influences movement at a fine‐temporal scale will be critical to the long‐term conservation of species impacted by climate change. The sedentary nature of non‐migratory species could increase some species risk of extirpation caused by climate change. We used Northern Bobwhite (*Colinus virginianus*; hereafter bobwhite) as a model to better understand the relationship between climate and the movement ecology of a non‐migratory species at a fine‐temporal scale. We collected movement data on bobwhite from across western Oklahoma during 2019–2020 and paired these data with meteorological data. We analyzed movement in three different ways (probability of movement, hourly distance moved, and sinuosity) using two calculated movement metrics: hourly movement (displacement between two consecutive fixes an hour apart) and sinuosity (a form of tortuosity that determines the amount of curvature of a random search path). We used generalized linear‐mixed models to analyze probability of movement and hourly distance moved, and used linear‐mixed models to analyze sinuosity. The interaction between air temperature and solar radiation affected probability of movement and hourly distance moved. Bobwhite movement increased as air temperature increased beyond 10°C during low solar radiation. During medium and high solar radiation, bobwhite moved farther as air temperature increased until 25–30°C when hourly distance moved plateaued. Bobwhite sinuosity increased as solar radiation increased. Our results show that specific climate variables alter the fine‐scale movement of a non‐migratory species. Understanding the link between climate and movement is important to determining how climate change may impact a species’ space use and fitness now and in the future.

## Graphical abstract

Because movement connects various aspects of an animal's life, research is needed to understand how climate influences movement to inform conservation efforts aimed at conserving species impacted by climate change. This study sought to understand how changes within specific climate variables alter the movement ecology of a non‐migratory animal. We conclude increased climate variability associated with climate change has the potential to alter the movement of many species, which may reduce survival and limit population connectivity in the future.

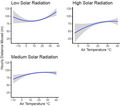

## 1 INTRODUCTION

Climate change continues to affect biodiversity and alter ecosystems across the globe by altering species distribution, increasing risk of extinction, and causing shifts in plant communities (McCarty, 2001; Murray et al., 2017). As such, understanding the effects of climate on animal behavior is critical to the conservation and management of wildlife (King, 2005; McCarty, 2001). Furthermore, the effects of climate change are predicted to affect species differently, increasing the need for more information on how different traits among species shape their responses (Tagliari et al., 2021). Climate is comprised of many different variables including air temperature, barometric pressure, solar radiation, wind, relative humidity, and precipitation that interact to create climate (Ahrens & Henson, 2016). As climate change continues, climate extremes (e.g., extreme heat or cold, drought, and floods) are projected to increase in frequency and intensity across the planet (Cohen et al., 2018; IPCC, 2021).

Many animals have specific behavioral strategies to cope with climate extremes (Cunningham et al., 2021; Melin et al., 2014; Pattinson & Smit, 2017). For example, some species adjust their immediate position on the landscape to seek cooler temperatures to mitigate extreme heat (Mason et al., 2017; Melin et al., 2014; Pattinson & Smit, 2017; Tanner et al., 2017). However, these strategies can have major consequences on the survival and population dynamics of species by reducing foraging efficiency, reproductive success, and an animal's ability to access resources (Pattinson & Smit, 2017; Tanner et al., 2017; van de Ven et al., 2020). Furthermore, increased climate extremes are expected to cause catastrophic population declines by increasing the difficulty for species to locate adequate amounts of food and cover, which can create resource bottlenecks (Maron et al., 2015). As climate change continues across the globe, understanding the role of climate on animal behavior is critical to conserving species affected by climate change.

One specific animal behavior, movement, combines various parts of an animal's life, including foraging, predator avoidance, and reproduction (Nathan et al., 2008). Changes in an animal's environment (Alston et al., 2020; Etzenhouser et al., 1998) and life history (Fies et al., 2002; Lenz et al., 2015) directly influence the movement ecology of animals. These changes alter the spatial and temporal arrangement of individuals across landscapes impacting survival (Somveille et al., 2015; Zollner & Lima, 2005), nutrient and energy flow within and across ecosystems (Earl & Zollner, 2014), gene flow (Clobert et al., 2001), and structural and distributional shifts in populations (Earl et al., 2016; Knowlton & Graham, 2010; Nathan et al., 2008). Movement can be split into two distinct behaviors (long‐distance dispersal and local movement; Earl et al., 2016; Rakowski et al., 2019), which can allow species to respond to changes in environmental conditions differently. For instance, some species respond to changes in environmental conditions by displacing long distances to more environmental‐benign areas (Nicholson et al., 2016; Somveille et al., 2015), while others remain in the same area all‐year round and withstand exposure to extreme climatic ets by utilizing specific habitat on the landscape (Alston et al., 2020; Carroll et al., 2015; Rakowski et al., 2019). Therefore, it is important to understand how the local movement of animals is impacted by climate as local movement directly influences the daily lives of animals by allowing them to accomplish various activities (e.g., foraging, predator avoidance, and reproductive duties) important for maintaining the individual as well as warrantying long‐term survival of a species (Geary et al., 2020; Hernández & Laundré, 2005; Precioso et al., 2020). Such knowledge has the potential to be used to conserve global biodiversity in the future as climate change continues to threaten ecosystems and biodiversity across the world (McCarty, 2001).

Non‐migratory species may be at a higher risk for extirpation due to climate change because they rarely disperse long distances (Earl et al., 2016; Jiguet et al., 2007; Townsend et al., 2003). Further, increased migratory diversity (i.e. movement plasticity) likely helps species that employ partial migratory strategies to be more resilient to environmental change than species that are purely sedentary (Gilroy et al., 2016). As such, non‐migratory species may be more exposed to climate variability and its potential impacts on fitness, particularly since many strategies used by species to combat climate extremes can negatively impact survival and reproductive success (Cunningham et al., 2021). In the future, climate change may intensify the thermal conditions of some landscapes leaving some animals without the ability to locate thermal refuge to survive extreme heat or potentially extending their stay in a thermal refuge such that it compromises their ability to adequately forage meet energy and nutrient demands (Carroll et al., 2016; Mason et al., 2017; Pattinson & Smit, 2017). Finally, because non‐migratory species typically depend on predictable resources within their home range, increased resource scarcity associated with climate change has the potential to threaten many non‐migratory species (Maron et al., 2015). However, it should be noted that not all species will be negatively impacted by climate change and that understanding the effect of climate change on a species is dependent on the context and the species (Murray et al., 2017; Tagliari et al., 2021). Given the increased likelihood of increased climate extremes in the future (IPCC, 2021), it is necessary we understand how climate influences the movement of non‐migratory species to better understand the effects of climate change on these species.

We studied the Northern Bobwhite (*Colinus virginianus*; hereafter, bobwhite) a non‐migratory Northern American bird (i.e., Galliforme) on the western edge of their distribution as a model to better understand the relationship between climate and the movement ecology of a non‐migratory species. Bobwhite are a declining non‐migratory species that typically live within 1 km of their natal site, yet have a broad geographic range across the eastern United States that extends westward into the Great Plains (Brennan et al., 2020; Townsend et al., 2003). Because of this, bobwhite frequently experience climate extremes on the western edge of their distribution, where periodic drought and extreme heat are common, making them an ideal species to study the role of climate on the movement ecology of non‐migratory animals (Arndt, 2003; Brennan et al., 2020; Carroll et al., 2017). Furthermore, recent advances in global positioning system (GPS) technology now allow bobwhite to be fitted with GPS tags (Cagnacci et al., 2010), allowing us to study their local movement at fine temporal and spatial scales (e.g., hourly timescales). Previous studies investigating the role of climate on animal movement have typically only analyzed movement at a broad temporal scale (i.e., daily movement; Garstang et al., 2014; Gong et al., 2020), but analyzing movement at a finer temporal scale allows us to better perceive more immediate changes in an animal's movement patterns across the day. In addition, many studies have only investigated how a specific climate variable (i.e., temperature) influences animal movement (Alston et al., 2020; Mason et al., 2017; Rakowski et al., 2019). These studies found that hotter air temperatures caused these species to become more sedentary and/or altered their position on the landscape (Alston et al., 2020; Mason et al., 2017; Rakowski et al., 2019). Given that climate is comprised of a variety of different variables (Ahrens & Henson, 2016), it is important to analyze movement at a fine temporal scale across multiple climate variables to better understand the role of climate on the movement of a non‐migratory species. Therefore, our objective was to understand how different climate variables affect the movement ecology of a non‐migratory species at a fine temporal scale (e.g., hourly timescales) by using bobwhite GPS data and meteorological data obtained from various environmental monitoring stations. Specifically, these data allowed us to investigate how air temperature, barometric pressure, solar radiation, relative humidity, average wind speed, average vector wind direction, and fractional water index (i.e., drought index) alter the movement characteristics (i.e., probability of movement, hourly distance moved and sinuosity) of a non‐migratory species throughout the day.

## 2 MATERIALS AND METHODS

### 2.1 Study areas

**FIGURE 1:**
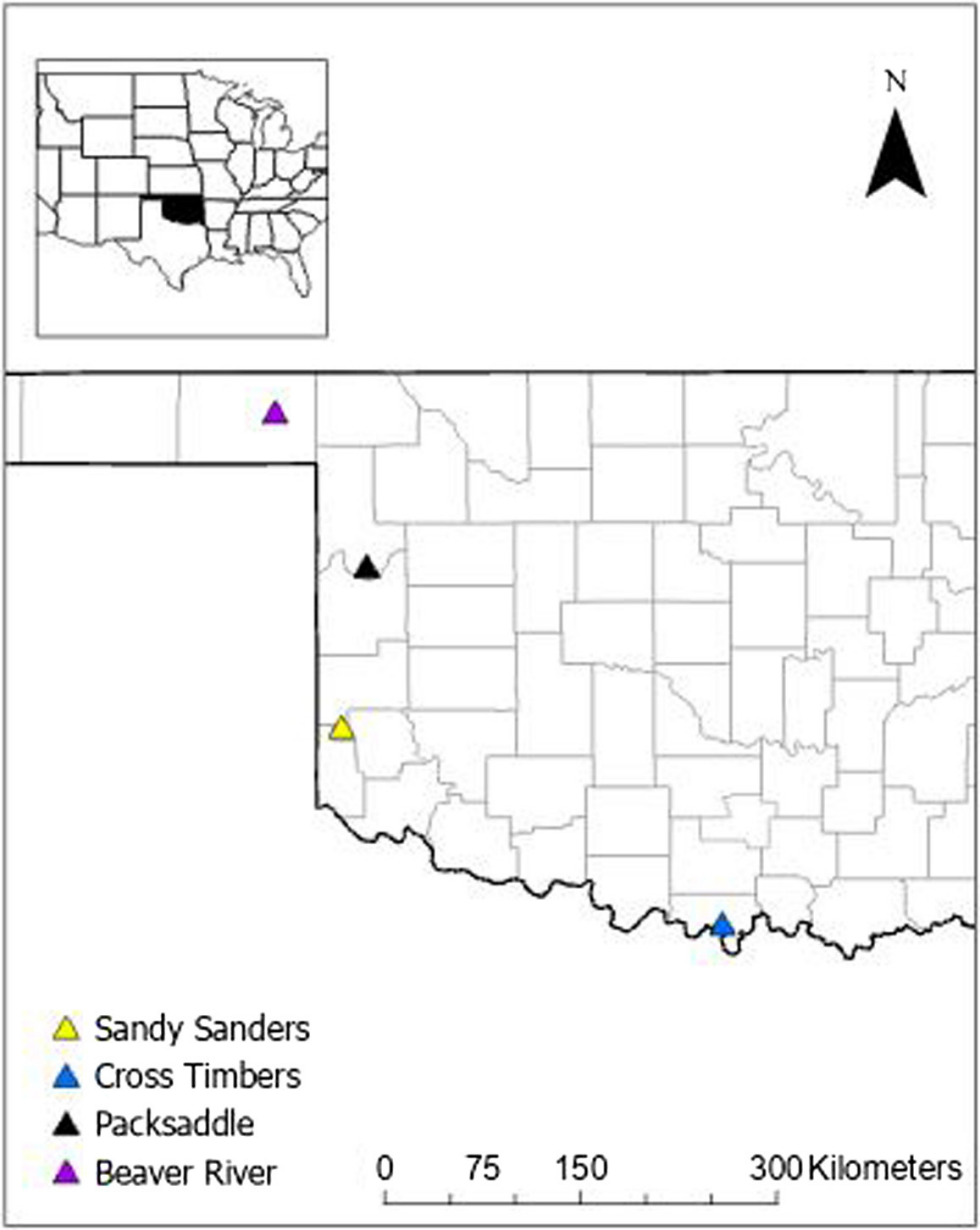
Location of the four study sites in Oklahoma where Northern Bobwhite (*Colinus virginianus*) were fitted with GPS technology and tracked during 2019–2020

We collected GPS data from bobwhite at the following wildlife management areas across western Oklahoma, USA: Cross Timbers (33.964043, −97.366169), Packsaddle (35.895249, −99.717387), Sandy Sanders (35.071182, −99.837630), and Beaver River (36.832998, −100.608260; Figure 1) from January 2019 to December 2020. These sites represent the wide range in climate that exists throughout western Oklahoma. During 2019–2020, air temperature ranged between −18.0 and 44.1°C across our sites with mean (±SE) air temperature being 15.3°C ± 0.1 (Brock et al., 1995; McPherson et al., 2007). Mean (±SE) annual rainfall across these sites during 2019–2020 was 833.6 mm ± 121.8, but ranged from 581.7 to 1165.9 mm (Oklahoma Climatological Survey, 2021). Dominant vegetation communities at these sites ranged from shrubland dominated grasslands to grassland savannas. Common tree species at these sites include eastern cottonwood (*Populus deltoides*) and post oak (*Quercus stellate*). Across these sites common shrub species include shinnery oak (*Quercus havardii*), sand sagebrush (*Artemisia filifolia*), and Chickasaw plum (*Prunus angustifolia*). Common herbaceous plants at these sites include Indiangrass (*Sorghastrun nutans*) and Buffalograss (*Bouteloua dactyloides*).

### 2.2 Data collection

We captured male and female bobwhite year‐around using Stoddard walk‐in funnel traps (Smith et al., 1981; Stoddard, 1931) baited with a mixture of cracked corn (*Zea mays*) and milo (*Sorghum bicolor*) at all four wildlife management areas. Individual birds were aged, sexed, and weighed and then fitted with a 7.2 g, solar‐powered GPS transmitter (Lotek, Wareham, United Kingdom; average location accuracy <15 m) that was attached by a backpack‐style harness made from 4.76‐mm tubular Teflon^®^ ribbon. If we trapped a covey, we only fitted a maximum of four individuals from that covey with transmitters. This was done to maintain an adequate number of individuals within a covey while maximizing the number of coveys with marked individuals as much as possible. Transmitters were only attached to bobwhite weighing ≥150 g to ensure that the transmitter weights did not exceed 5% of the bird's body weight (Bridge et al., 2011). If we trapped a covey with more than four individuals that passed this weight requirement, we randomly selected individuals that were fitted with transmitters. GPS transmitters were programmed to collect 18 hourly fixes per day between 0500 and 2200 central daylight savings time (CDT), but fewer hourly fixes occasionally occurred due to poor satellite transmission or low battery voltage. We did not sample between 2200 and 500 hours to conserve battery life. Overall, we fitted 338 (i.e., 188 males (120 juiles, 68 adults) and 150 females (97 juiles, 53 adults)) bobwhite with transmitters. All trapping and handling protocols were approved by Oklahoma State University Animal Care and Use Committee (ACUP AG‐18‐7).

Because climate consists of many different variables (e.g., air temperature, solar radiation, and relative humidity; Ahrens & Henson, 2016) we utilized the Oklahoma Mesonet, an extensive environmental monitoring network that collects meteorological data at fine temporal across each county in Oklahoma (Brock et al., 1995; McPherson et al., 2007). Previous studies show that animal movement can be influenced by different climate variables (e.g., air temperature; Alston et al., 2020; Gong et al., 2020; Rakowski et al., 2019). From January 2019 to December 2020 (bobwhite monitoring period), we acquired hourly weather data (air temperature, average wind speed, average vector wind direction, relative humidity, solar radiation, barometric pressure, and calibrated delta‐T; Table 1) from the following Mesonet stations: Beaver (Beaver WMA), Arnett (Packsaddle WMA), Erick and Elk City (Sandy Sanders WMA), and Burneyville (Cross Timbers WMA). We could not record instantaneous precipitation ets because Mesonet was unable to collect such data; however, collecting barometric pressure and calibrated delta‐T can provide an index for rainfall events (Ahrens & Henson, 2016; Illston et al., 2008 **)**. We standardized barometric pressure across all four sites by using the following equation to reduce barometric pressure to sea level (Keisan, 2018):

$$\begin{array}{c} \text{PRES}\;(\text{Reduced to Sea Level})=\\ p\times {\left(1‐\left(0.0065\times h\right)/\left(T^{\circ }C+273.15+0.0065\times h\right)\right)}^{‐5.257}, \end{array}$$

where *p* is barometric pressure, *h* is altitude, and *T* is air temperature.

**TABLE 1:** Description of climate variables collected from Beaver, Arnett, Erick, Burneyville, and Elk City mesonet stations located across western Oklahoma and those calculated from mesonet data during 2019–2020

Climate variable	Units	Collection specifics	Observed range 2019–2020
Air temperature	°C	1.5 m above ground	−18.0–44.1
Average wind speed	ms^−1^	2 m above ground, 5‐minute average	0–17.9
Average vector wind direction	degrees	10 m above ground, 5‐minute average	0–360
Relative humidity	%	1.5 m above ground	5.5–100.0
Solar radiation	Wm^−2^	–	0–1203.1
Barometric pressure	Mb	–	892.7–1013.4
Fractional water index		5 cm below ground	−0.03–1.04

Note

Calibrated Delta‐T was used to calculate fractional water index (Illston et al., 2008). Barometric pressure at each site was converted to sea level to standardize (Keisan, 2018).

We grouped average vector wind direction into the four cardinal directions based on their corresponding degrees. We used calibrated delta‐T to calculate fractional water index (FWI), a drought index, using the following equation (Illston et al., 2008):

$$\text{FWI}=\left(3.96^{\circ }C‐\text{Reference Temperature Difference}\right)/\left(3.96^{\circ }C‐1.38^{\circ }C\right).$$

### 2.3 Data analysis

We used Program R version 4.1.2 to perform these analyses (R Core Team, 2021). We excluded the first day of data collection for each bird to allow for acclimation to GPS transmitters. To account for GPS error, we removed all GPS fixes that were based on less than four acquired satellites or had an indicated dilution of precision >3.9. Following these corrections to the GPS fixes, average GPS error was confirmed to be <15 m (K. Andersson, Oklahoma State University, unpublished data). We also removed any duplicate fixes with the same timestamp. Because different movement metrics have been developed to describe different structural aspects of a movement path (consecutive relocations in a time series of geographic fixes), we analyzed two different movement metrics: hourly movement (displacement between two consecutive geographic fixes an hour apart) and sinuosity (form of tortuosity that determines the amount of curvature of a random search path; Almeida et al., 2010; Benhamou, 2004; Bovet & Benhamou, 1988; Seidel et al., 2018). We analyzed the data using a two‐step approach. First, we used hourly movement to investigate how different climate variables influence when bobwhite move and the hourly distance moved of actively moving bobwhite. Second, we used sinuosity to understand how different climate variables alter the sinuosity of actively moving bobwhite.

#### 2.3.1 Hourly movement

We used the R package “amt” to calculate hourly movement (Signer et al., 2019). We used the function *track_resample* to organize our movement data into hourly consecutive bursts across each individual and then used the functions *filter_min_n_burst*, and *steps_by_burst* to resample our entire dataset into a continuous series of 1‐h movements across each individual and to calculate hourly movement (Signer et al., 2019). Using hourly movement allowed us to understand how changes in specific climate variables alter the movement of a non‐migratory animal at a fine temporal scale. At the beginning timestamp for each hourly movement, we paired each hourly movement with the appropriate Mesonet data that aligned with the correct site and timestamp. Because of this, we selected the closest Mesonet station to the nearest WMA to pair the most appropriate Mesonet data to the GPS data of a specific site together according to the same timestamp. Mesonet stations were paired with the following WMA: Beaver (Beaver WMA), Arnett (Packsaddle WMA), Erick and Elk City (Sandy Sanders WMA), and Burneyville (Cross Timbers WMA). For Sandy Sanders WMA we used data from two Mesonet stations because we were unable to acquire calibrated delta‐T data from the nearest Mesonet station (Erick) to Sandy Sanders WMA. Because of this, all Mesonet data was paired with Sandy Sanders using Erick Mesonet station except calibrated delta‐T, which was acquired from Elk City Mesonet station. All Mesonet stations used for this study were located approximately 1.9–24.9 km from the nearest WMA. We used hourly movement to address two different questions using two separate analyses: probability of movement and hourly distance moved.

#### 2.3.2 Probability of movement

Our first analysis using hourly movement investigated how different climate variables influence when bobwhite move. To analyze the data, we utilized binomial distributed generalized linear mixed models using the R package “lme4” (Bates et al., 2015) and modeled the data using a binary response variable (movement or no movement) generated from the hourly movement data. We classified all hourly movements below the average GPS error rate (<15 m) as non‐movement (recorded as zero) and classified all hourly movements >15 m as movement (recorded as one). Within each model we included id nested in site as a random intercept to account for individual heterogeneity, potential pseudo‐replication, une sampling among individuals within each site and environmental differences at each site (Cady et al., 2021; Gillies et al., 2006). We removed any individuals that had <10 hourly movements because of data constraints when fitting a random effect structure within our models. We scaled each continuous independent variable using the scale function, which first centers the data of an independent variable by subtracting the variable mean from each specific data point of that variable and then scales that variable by dividing the centered data by their standard deviation. We scaled the data because differences in scale across the continuous independent variables caused challenges for models to converge. Scaling standardizes continuous variables on varying variable scales, which provides comparable model coefficients allowing easier model convergence.

For development and testing of our models, we used an *a priori* approach to determine the most appropriate models given our data (Burnham et al., 2011). A Pearson correlation test found no significant correlation (Nettleton, 2014) between independent variables included together in models (all *r* −.26 to .49). We evaluated one interaction based on previous research that indicated bobwhite broods select refuge sites that buffer against extreme heat caused by the interaction between air temperature and solar radiation (Carroll et al., 2015). In addition to these models, we assessed an additional model (time of day) to determine whether time of day alone better describes the relationship between probability of movement of bobwhite than any climate variable. We analyzed time of day using the beginning hour from each hourly movement. We quantified which model best supported the data by using Akaike Information Criterion for small sample sizes using the R package “bbmle” (Bolker & R Core Team, 2021). We considered models competitive if a model had a Δ AICc <2.0 (Symonds & Moussalli, 2011). We did not perform any model averaging because we simply wanted to use an AICc approach to better understand which climate variable or set of climate variables best describe the probability of movement of this species. Because of challenges interpreting the results from the best‐fit model associated with scaling the data, we calculated the relative movement frequency for each individual from our data across each air temperature (°C) value (i.e., whole number) and then graphed it continuously based on the best‐fit model. Because the best‐fit model consisted of an interaction, we parsed solar radiation (Wm^−2^) into three categories (low, medium, and high), representing the lower 25th, 25th–75th, and upper 75th percentiles of the data for graphing purposes.

#### 2.3.3 Hourly distance moved

The objective of our second analysis using hourly movement was to determine how different climate variables alter the hourly movement of bobwhite once individuals were moving. To analyze the data, we used gamma distributed generalized linear mixed models with log link functions using the R package “lme4” (Bates et al., 2015). The response variable associated with this analysis was hourly distance moved (m) of actively moving individuals using hourly movement data. Because of this, we removed all hourly movements below the average error rate of the GPS transmitters (<15 m). This removed any hourly movements that were sedentary from this analysis. Within each model, we included individual nested in site as a random intercept. Because air temperature (°C), barometric pressure (Mb), average wind speed (ms^−1^), and time of day (h) exhibited a quadratic relationship, these variables were fit with a quadratic polynomial term when present in a model (Ostertagová, 2012). Our approach to model development and testing was the same to our approach for analyzing probability of movement. We determined that there was no significant correlation (Nettleton, 2014) between independent variables included together in models (Pearson correlation test: all *r* −.25 to .45).

#### 2.3.4 Sinuosity

To understand how actively moving bobwhite change their sinuosity in response to different climate variables, we used the R package “amt” to calculate sinuosity (Signer et al., 2019). We used a sinuosity index over a straightness index for this study because we could not assume that the search behavior associated with these animals’ movement was mostly oriented (Almeida et al., 2010; Benhamou, 2004). As a path becomes more tortuous, sinuosity increases in value; however, as a path become straighter the value becomes closer to 0 (Duffy et al., 2011). We used the functions *track_resample* to organize our movement data into hourly consecutive bursts across each individual and then used the *filter_min_n_burst* to resample our entire dataset into a continuous series of 1‐h movements across each individual (Signer et al., 2019). At the top of each hour, we paired each GPS location to the appropriate Mesonet data the same way as hourly movement, which meant that we aligned the data together according to the correct site and timestamp. Because calculating sinuosity requires paths with multiple fixes, we were unable to analyze sinuosity at a one‐hour scale (Duffy et al., 2011). Therefore, we split each individual bird's data into continuous 3‐h paths. We calculated sinuosity for each 3‐h path using the function *sinuosity* (Signer et al., 2019). We also averaged each climate variable across each 3‐h path. We analyzed time of day using the beginning hour of each 3‐h path.

To analyze the data, we used linear mixed models with a log‐transformed response variable using the R package “lme4” (Bates et al., 2015) with a response variable of sinuosity (unitless) to develop models that investigate how different climate variables shape the sinuosity of bobwhite movements relative to a 3‐h path. We chose a linear mixed‐modeling approach with a log‐transformed response variable over a generalized mixed modeling approach with a log‐link function because sinuosity fit a log‐normal distribution, which led to challenges converging models when modeling sinuosity using gamma distributed generalized mixed models with a log‐link function. We removed paths where individuals moved less than the average GPS error rate (<15 m) because we were interested in analyzing the sinuosity of actively moving bobwhite. In addition, including paths of sedentary bobwhite led to unrealistic sinuosity values difficult to model. Within each model, we included a random intercept of individual nested in site. Because barometric pressure had a quadratic relationship, we fit it with a quadratic polynomial term when included in a model (Ostertagová, 2012). Our approach to model development and testing sinuosity was the same to our approach to model development and testing probability of movement and hourly distance moved. There was no significant correlation (Nettleton, 2014) between independent variables included together in models (Pearson correlation test: all *r* −.26 to .49).

## 3 RESULTS

### 3.1 Probability of movement

**FIGURE 2:**
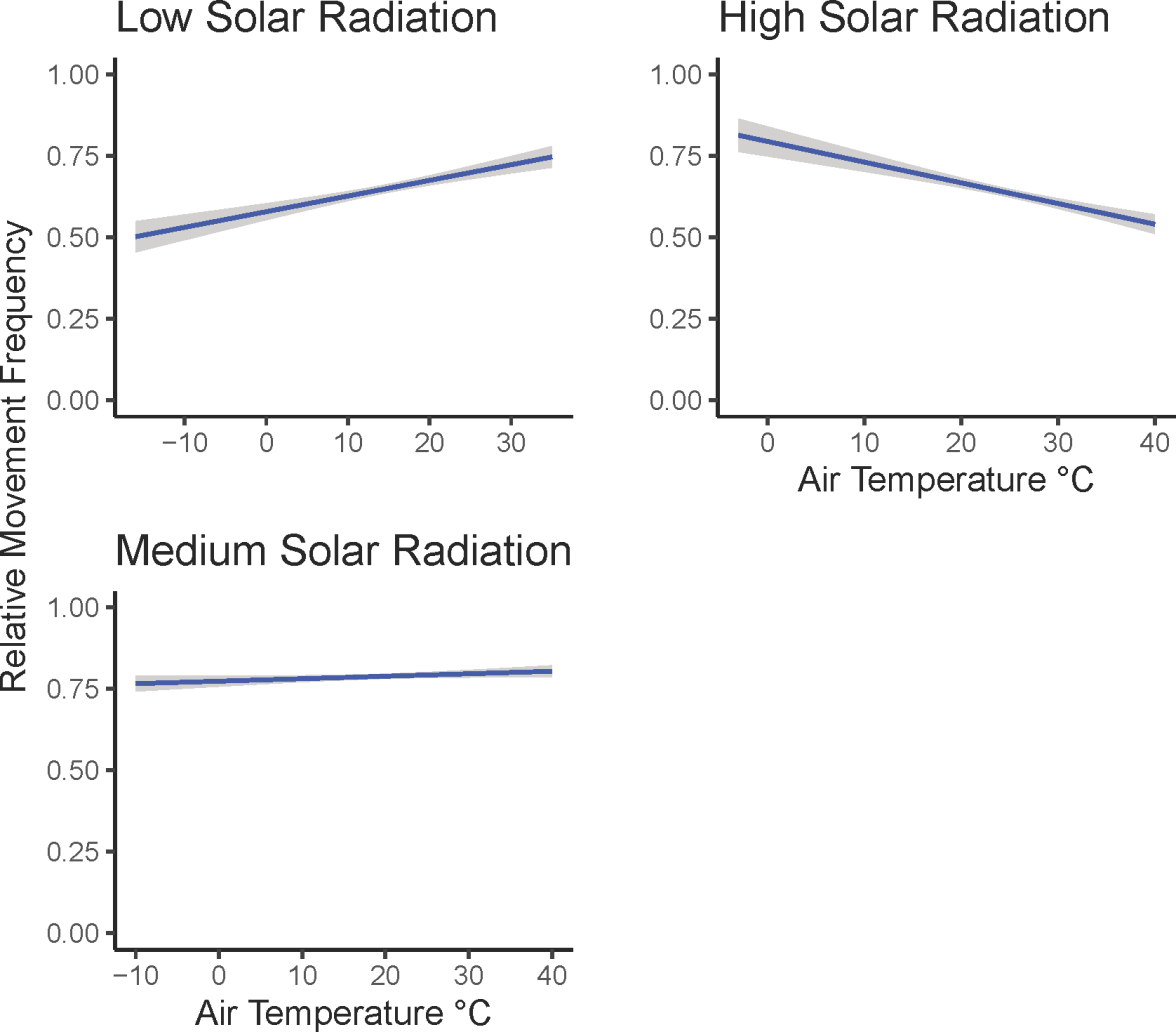
Observed relative movement frequency from Northern Bobwhite (*Colinus virginianus*) in western Oklahoma during 2019–2020 in response to the interaction between air temperature and solar radiation. Each regression line was fitted with a 95% confidence interval. For graphing purposes, we grouped solar radiation categorically as low (0–33.32 Wm^−2^), medium (33.33–666.82 Wm^−2^), and high (666.83–1203.12 Wm^−2^); which represents the lower 25th, 25th–75th, and upper 75th percentiles of the data

We analyzed 46,890 hourly movements from 283 bobwhite. Across sites, 45% of movements were at Packsaddle (*n* = 21,027), 28% at Beaver River (*n* = 13,311), 18% at Cross Timbers (*n* = 8434), and 9% at Sandy Sanders (*n* = 4118). In our dataset, mean (±SE) hourly movement was 47.5 m ± 0.40 with a range from 0 to 1882.5 m. We investigated 16 models to understand how different climate variables alter the probability of movement of a non‐migratory bird. The best‐fit model was the interaction between air temperature and solar radiation (Table 2; marginal *R* ^2^ = 0.026, conditional *R* ^2^ = 0.207) suggesting that probability of movement was affected by the interaction effects of air temperature and solar radiation (Table 3). When calculating relative movement frequency across our data, we determined that the interaction between air temperature and solar radiation influenced the relative movement frequency of bobwhite differently. During low solar radiation, relative movement frequency increased as air temperature increased (Figure 2). However, when high solar radiation occurred relative movement frequency decreased as air temperature increased (Figure 2). The interaction between air temperature and medium solar radiation had little effect on the relative movement frequency of bobwhite.

**TABLE 2:** Model comparison table showing the top 5 best fit models of the 16 models that we evaluated to investigate how different climate variables altered probability of movement, hourly distance moved, and sinuosity (3‐h path) of Northern Bobwhite in western Oklahoma during 2019–2020

Model variables	df	Log‐likelihood	AICc	dAICc	AICc weight
Probability of movement					
TAIR*SRAD	5	−29209.6	58429.2	0.0	1
TAIR + SRAD	4	−29555.1	59118.2	689.1	<0.001
Time of day	3	−29611.5	59228.9	799.8	<0.001
SRAD	3	−29703.3	59412.2	983.1	<0.001
FWI + TAIR	4	−29727.3	59462.6	1033.4	<0.001
Hourly distance moved					
TAIR*SRAD + TAIR^2^*SRAD	8	−128714.2	257444.3	0.0	0.988
TAIR + TAIR^2^ + SRAD	6	−128720.5	257453.1	8.8	0.012
Time of day + Time of day^2^	5	−128767.4	257544.8	100.4	<0.001
SRAD	4	−128783.5	257575.1	130.8	<0.001
PRES + PRES^2^ + AWDIR + AWSP + AWSP^2^	10	−128821.4	257662.9	218.5	<0.001
Sinuosity					
SRAD	4	−12756.3	25520.6	0.0	0.942
TAIR + SRAD	5	−12758.6	25527.1	6.5	0.036
FWI	4	−12760.9	25529.7	9.2	0.010
TAIR*SRAD	6	−12759.3	25530.5	10.0	0.007
Null	3	−12762.6	25531.2	10.6	0.005

Abbreviations: AWDIR, average vector wind direction; AWSP, average wind speed; FWI, fractional water index; PRES, barometric pressure; SRAD, solar radiation; TAIR, air temperature.

**TABLE 3:** Model output from each top model modeling probability of movement, hourly movement, and sinuosity (3‐h path) of Northern Bobwhite in western Oklahoma during 2019–2020

Fixed effects	Estimate	Std. error	*t*‐Value	*p*‐Value	Random effect (SD)
Probability of movement					
Intercept	0.044	0.060	0.748	.454	0.955
TAIR	0.189	0.016	11.903	<.001	
SRAD	−0.158	0.013	−12.495	<.001	
TAIR*SRAD	−0.308	0.012	−26.049	<.001	
Hourly distance moved					
Intercept	4.482	0.022	202.002	<.001	0.316
TAIR	0.085	0.008	10.477	<.001	
TAIR^2^	−0.006	0.005	−1.087	.277	
SRAD	−0.092	0.007	−12.843	<.001	
TAIR:SRAD	−0.013	0.007	−1.906	.057	
TAIR^2^:SRAD	−0.015	0.005	−3.040	.002	
Sinuosity					
Intercept	−1.729	0.018	−96.100	<.001	0.138
SRAD	0.057	0.013	4.420	<.001	

Note

For the binomial movement model a *z*‐value not a *t*‐value was calculated.

Abbreviations: SRAD, solar radiation; TAIR, air temperature.

### 3.2 Hourly distance moved

**FIGURE 3:**
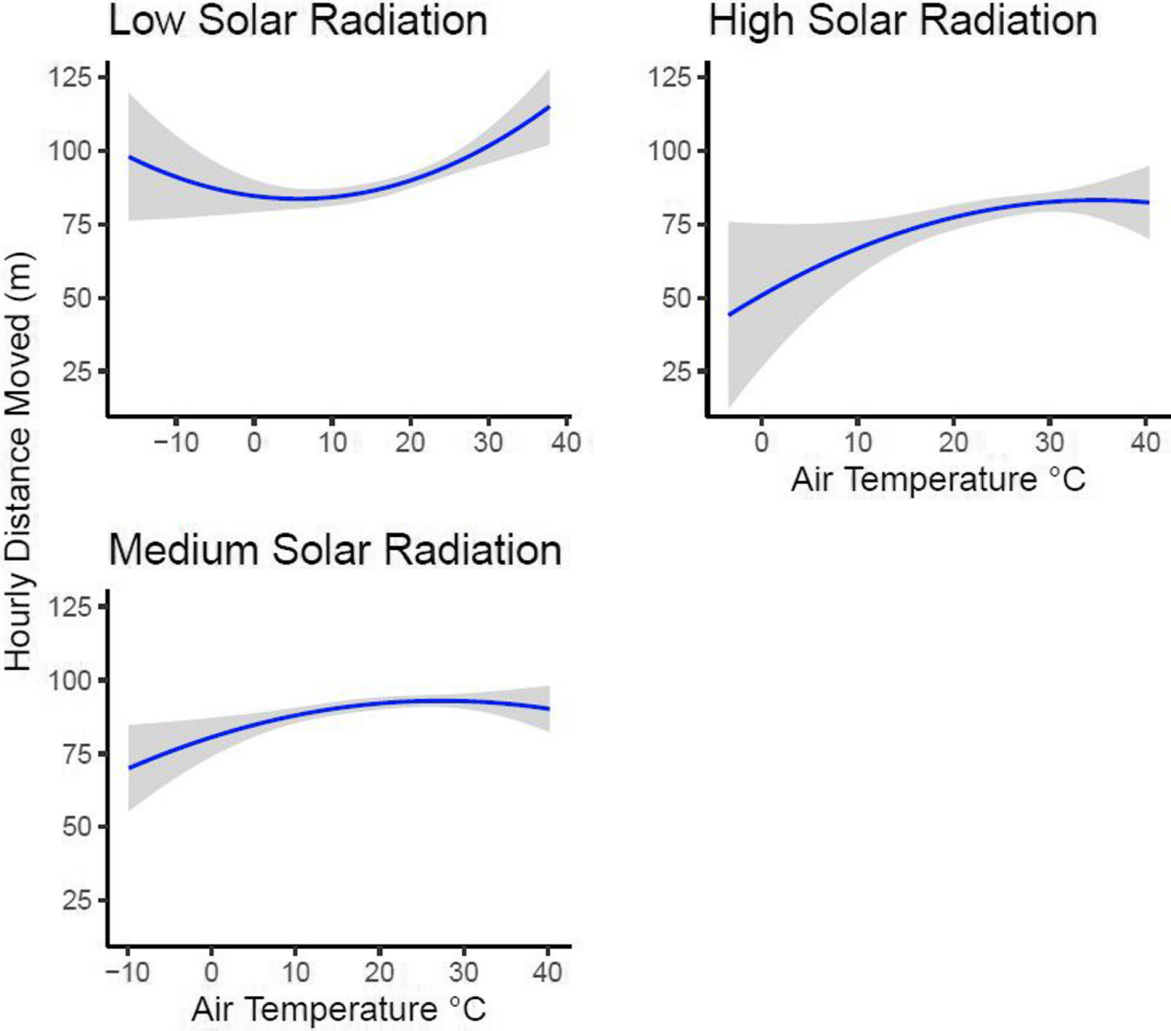
The relationship between the hourly distance moved of Northern Bobwhite (*Colinus virginianus*) in western Oklahoma during 2019–2020 once an individual was moving in response to the interaction between solar radiation and air temperature. Each regression line was fitted with a 95% confidence interval and a polynomial because air temperature exhibited a quadradic relationship. For graphing purposes, we grouped solar radiation categorically as low (0–79.74 Wm^−2^), medium (79.75–602.33 Wm^−2^), and high (602.34–1203.12 Wm^−2^); which represents the lower 25th, 25th–75th, and upper 75th percentiles of the data

For this part of the study, we analyzed 23,911 hourly movements from 242 actively moving bobwhite, with 47% of the movements from Packsaddle (*n* = 11,233), 28.5% from Beaver River (*n* = 6808), 16.5% from Cross Timbers (*n* = 3923), and 8% from Sandy Sanders (*n* = 1947). Mean hourly movement (±SE) of actively moving bobwhite was 87.8 m ± 0.6. We investigated 16 models to determine how different climate variables affect the hourly distance moved of actively moving bobwhite. Similar to probability of movement, the best fit model was the interaction between air temperature and solar radiation (Table 2; marginal *R* ^2^ = 0.006, conditional *R* ^2^ = 0.064). Overall, bobwhite moved shorter distances as the interaction between air temperature and solar radiation increased (Table 3, Figure 3). Graphing showed that different solar radiation intensities alter how air temperature influences hourly distance moved. For instance, during low solar radiation, bobwhite moved farther as air temperature increased e when air temperature increased beyond 30°C (Figure 3). During medium and high solar radiation, bobwhite moved farther as air temperature increased until 25–30°C when hourly distance moved plateaued (Figure 3).

### 3.3 Sinuosity

**FIGURE 4:**
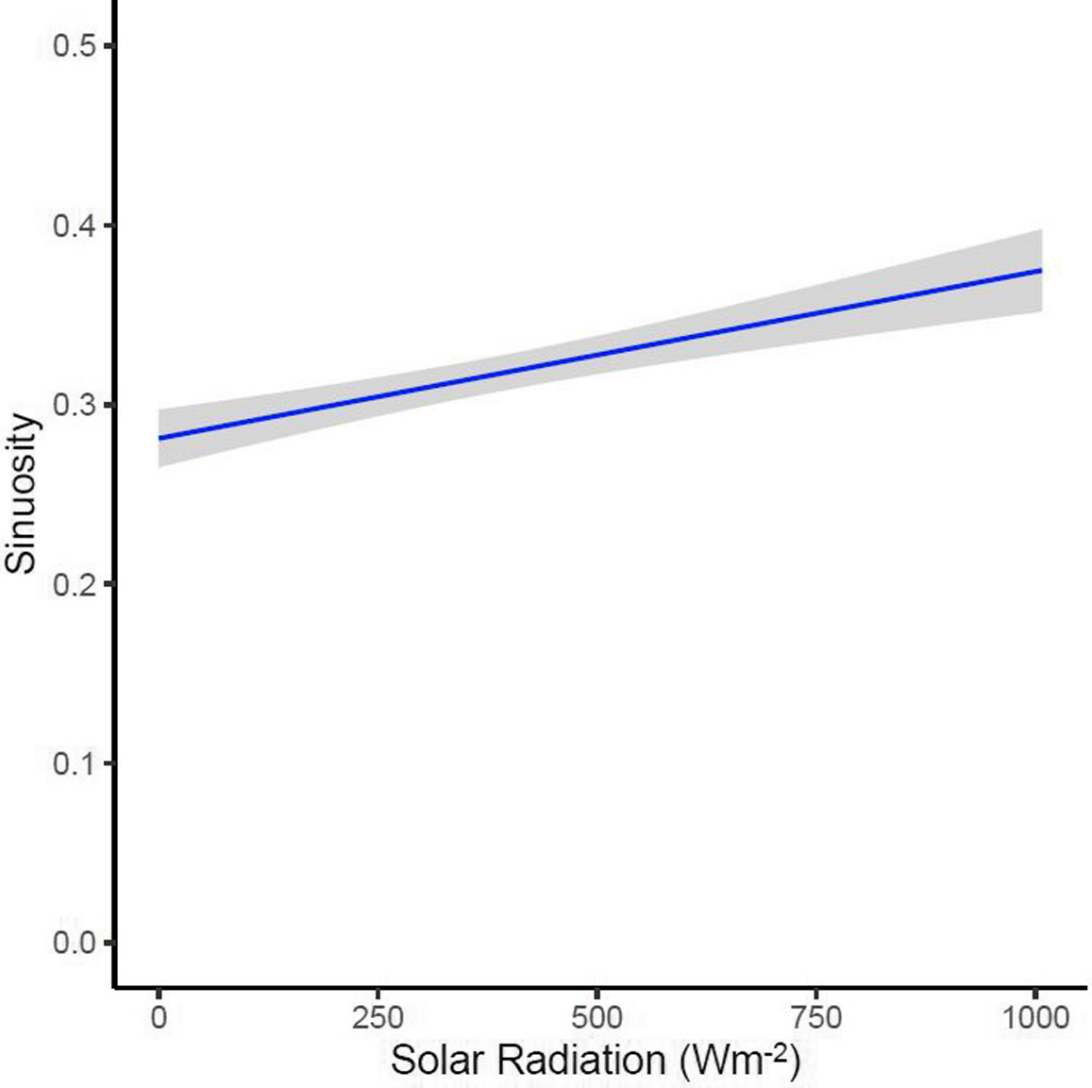
The relationship between the sinuosity of Northern Bobwhite (*Colinus virginianus*) in western Oklahoma during 2019–2020 relative to a 3‐h path once an individual was moving in response to solar radiation. Fitted along the regression line was a 95% confidence interval. As a path becomes more tortuous, sinuosity increases in value; however, as a path becomes straighter the value becomes closer to 0 (Duffy et al., 2011)

We analyzed 8193 3‐h paths from 181 actively moving bobwhite. Forty‐seven percent of the movements occurred at Packsaddle (*n* = 3824), 27% at Beaver River (*n* = 2233), 18% at Cross Timbers (*n* = 1496), and 8% at Sandy Sanders (*n* = 640). Mean (±SE) sinuosity of actively moving bobwhite relative to a 3‐h path was 0.3 ± 0.01 with a range of 0.0005–12.0. Similar to previous analyses, we evaluated 16 models to determine how the sinuosity of an actively moving non‐migratory animal is influenced by different climate variables. The model that best described the data only included a single variable, solar radiation (Table 2; marginal *R* ^2^ = 0.002, conditional *R* ^2^ = 0.017). The 3‐h paths of bobwhite became more tortuous as solar radiation intensity increased (Table 3, Figure 4).

## 4 DISCUSSION

Our study aligns with a growing body of research that indicates that specific climate variables that comprise climate influence animal movement (Alston et al., 2020; Bourgoin et al., 2011; Gong et al., 2020; Rakowski et al., 2019). Thus, increased climate variability associated with climate change has the potential to alter the movement of many species (IPCC, 2021; Thornton et al., 2014). Because movement connects various activities that influence an animal's survival, shifts in movement pattern may have long‐term impacts on the survival of animals by negatively influencing resource acquisition, survival, and population connectivity (Earl et al., 2016; Nathan et al., 2008; Zollner & Lima, 2005). This study highlights the influence of air temperature and solar radiation on the movement of a non‐migratory animal. Further, our study suggests that these climate variables may better describe fine‐scale movement patterns than simply time of day. Previous studies show that these climate variables alter animal movement and survival (Alston et al., 2020; Hovick et al., 2015; Lenarz et al., 2009; Rakowski et al., 2019). Our study went further by investigating how the interaction between these two climate variables influences movement. Because climate is comprised of multiple variables influencing each other (Ahrens & Henson, 2016), it should not be surprising that some animals alter their movement in response to interactions between different climate variables. In addition, bobwhite were most sedentary during extreme cold and heat. Previous studies show that temperature extremes can limit movement and alter an animal's position on the landscape, leading to increased mortality in some animals potentially caused by limiting their ability to access specific areas of the landscape reducing resource availability (Aublet et al., 2009; Carroll et al., 2015; Melin et al., 2014; Tanner et al., 2017). Because non‐migratory species typically rely on predictable resources within a fixed home range (Maron et al., 2015), our findings suggest that increased climate extremes caused by climate change could impact the survival of many non‐migratory species, especially if it leads to animals becoming more sedentary and having less access to resources (IPCC, 2021). Some suggest that some species may have to shift their activity to different portions of the day in the future to survive increased climate extremes (Aublet et al., 2009). Further, species with a higher level of migratory diversity across individuals might be better able to cope with unpredictable environmental conditions in the future (Gilroy et al., 2016). Future research is needed to better understand the ability for species to shift their behavior during climate extremes to cope with ongoing climate change.

Changes in movement in response to the interaction between air temperature and solar radiation may reflect behavioral tradeoffs associated with increased hyperthermia risk caused by extreme heat (≥30°C) and increased solar radiation intensity (Boyles et al., 2011; Cunningham et al., 2021; Norris & Kunz, 2012). Some animals adjust their behavior to lower hyperthermia risk by reducing their movement (Rakowski et al., 2019), locating thermal refuge to limit thermal stress (Alston et al., 2020; Carroll et al., 2015), adjusting foraging behavior (Pattinson & Smit, 2017), and changing their posture (Maloney et al., 2005; Norris & Kunz, 2012). However, these behavioral adjustments can be costly (Cunningham et al., 2021), as increased behavioral thermoregulation caused by extreme heat can reduce foraging efficacy and reproduction success (Cunningham et al., 2013; Pattinson & Smit, 2017; van de Ven et al., 2020). Such implications could have lasting effects on the ability for species across the globe to persist (Mason et al., 2017; Pattinson & Smit, 2017). Climate change will likely cause many species to experience increased extreme heat in the future and exacerbate these concerns (Cunningham et al., 2021; IPCC, 2021). Our findings show that during the day bobwhite become more sedentary and move shorter distances during extreme heat, suggesting that the ability for many non‐migratory species to tolerate extreme heat and adapt to global change could be severely hindered by climate change (Jiguet et al., 2007).

Many studies have focused on understanding how extreme heat influences animal behavior (Carroll et al., 2017; Cunningham et al., 2021; van de Ven et al., 2020). However, climate change can also cause extreme cold during winter (Cohen et al., 2018), suggesting that other conditions beyond extreme heat could negatively impact animals too. Some animals reduce their activity during extreme cold, likely to conserve energy as extreme cold can cause increased hypothermia risk in animals (Cotton & Parker, 2000; Norris & Kunz, 2012). These challenges coupled with limited resources caused by extreme cold can reduce survival causing high mortality in some animals (Maron et al., 2015; Tanner et al., 2017). Our findings suggest that during the day bobwhite are most sedentary when air temperatures are ≤0°C during certain solar radiation intensities. However, air temperatures ≤0°C rarely occurred during this study limiting our ability to infer more from this trend. Additional studies in colder climates could be useful to better understand the impacts of extreme cold on animal movement.

We determined that solar radiation influences the sinuosity of a non‐migratory animal's 3‐h path. Given that many animals require thermal refuge to tolerate extreme heat (Carroll et al., 2015; Melin et al., 2014; Rakowski et al., 2019), demands to locate thermal refuge during periods of extreme heat could have caused the movement paths of bobwhite to become more sinuous. Despite this, solar radiation only caused bobwhite movement paths to become slightly more sinuous suggesting that the relationship between solar radiation and the sinuosity of bobwhite movement at a 3‐h temporal scale is weak. Nevertheless, climate change is predicted to reduce the ability for some animals to locate thermal refuge on the landscape by homogenizing the thermal landscape (Carroll et al., 2016). Because of this, increased climate variability in the future could increase the sinuosity of animals if locating thermal refuge becomes more difficult (Carroll et al., 2016). Furthermore, changing the temporal scale of an animal's movement path can yield different results (Kay et al., 2017). In addition, collecting GPS data at finer resolutions (e.g., movement data every 5 min) could have yielded a stronger relationship; however, our GPS transmitters were unable to acquire data at this interval. It is possible that analyzing sinuosity at a broader temporal scale and by using higher resolution GPS data could have yielded a stronger relationship with solar radiation. Future research should take advantage of advancements in technology to better understand the role of different climate variables on the sinuosity of an animal.

As global climate change continues, increasing the intensity and frequency of extreme heat and precipitation (IPCC, 2021), there is a growing need to understand how specific climate variables alter animal movement to better determine how to conserve species impacted by climate change. Our results add to the growing body of literature on this topic (Alston et al., 2020; Aublet et al., 2009; Bourgoin et al., 2011; Gong et al., 2020). Our findings show that the interaction between air temperature and solar radiation or simply solar radiation influence the fine‐scale movement of a non‐migratory animal. Because of this, increased climate variability caused by climate change may alter movement patterns and constrain the movement of animals in the future. However, these changes have the potential to favor some generalist species (Tagliari et al., 2021). For species impacted by increased climate variability, shifts in movement patterns may reduce an individual's ability to breed successfully disrupting population dynamics of a species (Cunningham et al., 2021; Mason et al., 2017; van de Ven et al., 2020). Because changes in movement patterns influence the connectivity of individuals and populations (Knowlton & Graham, 2010; Nathan et al., 2008), shifts in movement at fine‐temporal scales caused by increased climate extremes may have negative consequences for the long‐term viability of populations (Inoue and Berg, 2017; Murray et al., 2017). Furthermore, increased extreme heat or cold may render large portions of the landscape unsuitable for species that require adequate thermal refuge (Carroll et al., 2016; Tanner et al., 2017). Our study highlights the importance of understanding how different climate variables influence the movement of a non‐migratory bird at a fine‐temporal scale. Better knowledge determining what drives the fine‐scale movement patterns of a specific species is vital to decipher how climate change and other forms of environmental change are already impacting species now and in the future.

## CONFLICT OF INTEREST

None declared.

## AUTHOR CONTRIBUTIONS

**Landon K. Neumann:** Conceptualization (equal); Formal analysis (lead); Writing – original draft (lead); Writing – review & editing (lead). **Samuel D. Fuhlendorf:** Conceptualization (equal); Formal analysis (supporting); Funding acquisition (equal); Methodology (equal); Project administration (equal); Writing – original draft (supporting); Writing – review & editing (supporting). **Craig D. Davis:** Conceptualization (equal); Formal analysis (supporting); Funding acquisition (equal); Methodology (equal); Project administration (equal); Writing – original draft (supporting); Writing – review & editing (supporting). **Shawn M. Wilder:** Conceptualization (equal); Funding acquisition (equal); Methodology (equal); Writing – original draft (supporting); Writing – review & editing (supporting).

## Data Availability

The datasets used in this study are publicly available in the Dryad Digital Repository (https://doi.org/10.5061/dryad.n02v6wx0d).
