# Corrigendum

**DOI:** 10.1002/ece3.9834

**Published:** 2023-02-14

In the article by Neumann et al. (2022), the middle initial of the third author was incorrect. The article has been corrected online, so author byline now reads as:

Landon K. Neumann | Samuel D. Fuhlendorf | Craig A. Davis | Shawn M. Wilder

The authors apologize for the error.
